# Multipole Excitations and Nonlocality in 1d Plasmonic Nanostructures

**DOI:** 10.3390/nano13081395

**Published:** 2023-04-18

**Authors:** Anatoliy V. Goncharenko, Vyacheslav M. Silkin

**Affiliations:** 1 V.E. Lashkaryov Institute of Semiconductor Physics, Nauky Ave. 41, 03028 Kyiv, Ukraine; 2 Donostia International Physics Center (DIPC), Paseo de Manuel Lardizabal 4, 20018 San Sebastian, Spain; 3 Departamento de Polímeros y Materiales Avanzados: Física, Química y Tecnología, Facultad de Ciencias Químicas, Universidad del País Vasco (UPV/EHU), 20080 San Sebastian, Spain; 4 IKERBASQUE, Basque Foundation for Science, 48013 Bilbao, Spain

**Keywords:** plasmonic nanostructures, multipoles, nonlocality, metamaterials, transfer-matrix method

## Abstract

Efficient simulation methods for taking nonlocal effects in nanostructures into account have been developed, but they are usually computationally expensive or provide little insight into underlying physics. A multipolar expansion approach, among others, holds promise to properly describe electromagnetic interactions in complex nanosystems. Conventionally, the electric dipole dominates in plasmonic nanostructures, while higher order multipoles, especially the magnetic dipole, electric quadrupole, magnetic quadrupole, and electric octopole, can be responsible for many optical phenomena. The higher order multipoles not only result in specific optical resonances, but they are also involved in the cross-multipole coupling, thus giving rise to new effects. In this work, we introduce a simple yet accurate simulation modeling technique, based on the transfer-matrix method, to compute higher-order nonlocal corrections to the effective permittivity of 1d plasmonic periodic nanostructures. In particular, we show how to specify the material parameters and the arrangement of the nanolayers in order to maximize or minimize various nonlocal corrections. The obtained results provide a framework for guiding and interpreting experiments, as well as for designing metamaterials with desired dielectric and optical properties.

## 1. Introduction

The electric dipole approximation is commonly applied to study optical properties of various materials and structures. Generally, any nonmagnetic structure can be considered as an array of coupled discrete electric dipoles, while its electromagnetic properties can be explained in terms of the interference of the related electric dipole (ED) fields. This idea is at the heart of the well-known simulation technique, known as the coupled dipole (or discrete-dipole) approximation [1]. However, being computationally efficient, the coupled dipole approximation may provide no insight into the underlying physics. At the same time, electric and magnetic multipoles in nanomaterials are known to be responsible for a variety of optical phenomena, including artificial magnetism, additional extraordinary waves, negative refraction, giant electromagnetic chirality, enhanced spontaneous emission, superscattering, cloaking, etc.

Involving higher multipoles in materials and nanostructures for the description of their optical properties often becomes crucial to achieve desired functionalities [2]. As examples of more recent contributions to the field, the works on the generalized Kerker effect [3], topological Weyl metamaterials (MMs) [4], and Pancharatnam–Berry metasurfaces [5] can be mentioned. In fact, various nanostructures with a complex arrangement could give rise to enhanced or suppressed particular multipole excitations, offering new techniques to control their optical properties. Recently, a good review on multipole concepts with emphasis on achieving high directivities from radiating and scattering systems was given by Ziolkowski [6].

In homogeneous media, Raab and de Lange considered symmetry constraints for the constitutive relations, taking multipolar effects into account [7], and recapitulated and further developed the multipole theory [8,9]. They classified long-wavelength radiation effects that involve different multipole orders in the expansions of the displacement D and of the magnetic field H. Their hierarchy of the magnitudes of multipole contributions to the radiation–matter interaction is as follows: electric dipole ≫ electric quadrupole + magnetic dipole ≫ electric octopole + magnetic quadrupole and so on.

The literature on the role of multipole excitations in plasmonic nanosystems is abundant. So, higher-order multipolar modes are known to interfere with the broad dipole mode giving rise to high-order Fano resonances with asymmetric profile [10]. Cho et al. [11] considered the nonlocal effective response of MM consisting of a pair of coupled metal bars using the formalism of the effective permittivity and effective permeability. As they found, the contribution of the electric quadrupole (EQ) to the effective permeability can be comparable to that of the magnetic dipole (MD). Alu and Engheta [12] dealt with infinite linear chains of plasmonic nanoparticles, taking their quadrupolar resonance into account. They showed that under specific conditions, the quadrupolar response of a plasmonic nanoparticle can be dominant. Besides, they concluded that despite the narrow bandwidth of the individual quadrupolar resonance, the overall bandwidth of chain guidance can be large due to strong coupling among the nanoparticles. Grahn et al. [13], dealing with arrays of nanoscatterers, introduced a multipole decomposition of the electric currents in the scatterers and connected it to the classical multipole expansion of the scattered field. They found, in particular, that completely different multipoles can produce indistinguishable electromagnetic fields. Silveirinha [14] derived the boundary conditions for the fields at interfaces of media using the so-called quadrupolar media approximation, which takes into consideration only electric dipolar, magnetic dipolar, and electric quadrupolar excitations. Wells et al. [15] analyzed the electric field enhancement in a plasmonic conical nanorod array with an account for second-order nonlocal corrections to its effective permittivity tensor.

Lim et al. [16] applied a multipolar decomposition to the analysis of natural modes in complimentary MM structures and showed that the magnetic quadrupole (MQ) can give rise to the linewidth narrowing of the Fano resonance. Dirdal et al. [17] showed that the electric octopole (EO)-MQ term can be of the same order of magnitude as the MD and EQ terms in MMs. Babicheva and Evlyukhin [18] considered optical properties of subwavelength spherical nanoparticles and their periodic arrays with an emphasis on the MQ response. In particular, they concluded that under certain conditions the effective polarizabilities of the ED and MQ, taking multipole coupling into account, can be significantly reduced. Within the multipolar framework, Mun et al. [19] introduced the higher-order dynamic polarizability tensors, which can accurately represent anisotropic meta-atoms with higher-order multipole transitions. Liu et al. [20] showed that in double-Dirac-cone PCs with the electric field along the optical axis, quadrupole resonances contribute to the effective permittivity. Rahimzadegan et al. [21] dealt with periodic metasurfaces (2d arrays) and derived expressions for their optical response using a multipole expansion for fields including dipolar, quadrupolar, and octopolar terms. As an illustration, they applied the developed approach to a fully diffracting metagrating, a polarization filter, and Huygens’ metasurface.

One-dimensional plasmonic gratings attract the attention of physicists, as they can exhibit a number of interesting phenomena, such as, e.g., multiband absorption [22] or plasmonically induced transparency [23,24]. At the same time, despite a few efforts, the effect of multipole excitations on the nonlocal permittivity tensor remains poorly studied even in the simplest case of 1d MMs. So, the issue of the relationship between the unit cell geometry and the nonlocal response has not been addressed yet. In the present study, we take a step forward to assess the relative contributions of various nonlocal corrections to the nonlocal effective permittivity. As the corrections cannot be obtained in analytical form, our approach relies primarily on numerical simulations. In particular, it involves calculating the nonlocal permittivity with the use of the transfer matrix method (TMM), which is especially efficient for 1d nanostructures.

## 2. Geometry and Assumptions

**Figure 1:**
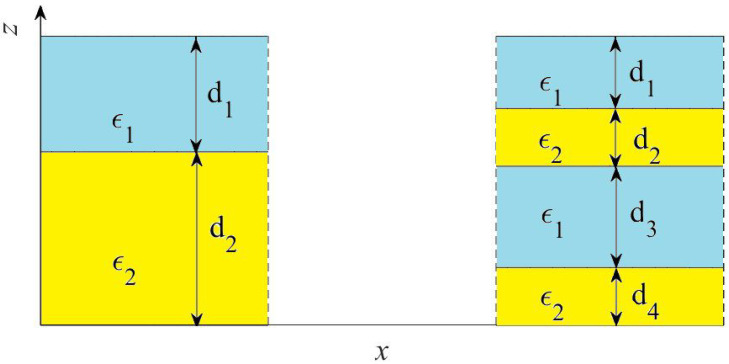
The sketches of the unit cell of two microgeometries under consideration: two-layer (**left panel**) and four-layer (**right panel**) microgeometries.

The nanostructures under consideration are assumed to be infinite in the *x*-*y* plane and periodic in the *z*-direction (see Figure 1). This allows us to avoid dealing with the violation of Maxwell boundary conditions resulted from nonlocality [25]. Furthermore, the following assumptions were made.

(1) We deal with nonmagnetic, homogeneous, isotropic dielectric, and plasmonic layers. Although multipolar excitations can occur in all-dielectric nanostructures due to retardation effects, this usually takes place at sizes where the long-wavelength multipole expansion is no longer accurate. In contrast, in plasmonic multilayered nanostructures strong optical nonlocality can occur for moderate size unit cells as a result from excitation of surface plasmon polaritons (SPPs). Currently, plasmonic MMs offer rich opportunities to control the light intensity and polarization, as well as the near-field heat transfer and local density of states on the subwavelength scale [26,27].
(2) We neglect intrinsic nonlocality of constituent layers. It means that the vectors of the electric displacement and magnetic fields **D** and **H** at any point of space can be written in terms of the spatially averaged electric and induction fields, **E** and **B**, at the same point. It is necessary to keep in mind that in some cases, especially for thin metal layers and gaps, the local response model for their permittivity may become inaccurate [28,29,30].
(3) To reduce the number of material parameters, we deal only with two-phase MMs. It implies that if the filling factors of the constituents 1 and 2 are $f_{1}$ and $f_{2}$, respectively, it is always $f_{1}+f_{2}=1$. At the same time, multiphase MMs with arbitrary number of different phases within unit cell can be studied using our technique.
(4) For simplicity, only microgeometries with two-layer and four-layer unit cells are considered as shown in Figure 1. We note that any three-layer microgeometry cannot be realized when dealing with 1d two-phase MMs.
(5) Herein, we limit ourselves to considering only the fundamental mode, which propagates along the interfaces ($k_{z}=0$) with the electric field, oriented along the optical axis (TM polarization). This means that we consider only one diagonal component ($\epsilon_{\|}$) of the nonlocal effective permittivity tensor ${\tilde{\epsilon }}_{ij}=diag\left(\epsilon_{\|},\epsilon_{⊥}\right)$. Thus, in what follows we implicitly assume that $\tilde{\epsilon }\equiv \epsilon_{\|}$. This choice is not accidental. It is motivated by the fact that this component can vary in wide limits, that gives rise to numerous potential applications [31,32,33,34].

Due to the simplifying assumptions made, the determination of the nonlocal effective permittivity becomes straightforward. In principle, this allows one to evaluate the nonlocal corrections of any order. In this study, we restrict ourselves by magnetic dipole and electric quadrupole, as well as magnetic quadrupole, electric octopole, and higher-order terms, up to the eighth order term in the wavenumber expansion.

## 3. Formalism and Techniques Used

According to the classical electrodynamics theory [35], for a nonlocal medium, the nonlocal permittivity ϵ(ω,k) can be introduced to relate the averaged electric field $E_{\mathrm{av}}$ and the generalized electric displacement vector $D_{g}=E_{\mathrm{av}}+4\pi P_{g}$ via $D_{g}=\epsilon \left(\omega ,k\right)E_{\mathrm{av}}$, where $P_{g}$ is the generalized polarization density vector. In what follows, we assume a harmonic time dependence exp(iωt) for all the fields. The generalized polarization density can be written as

(1) $$P_{g}=P_{d}+\frac{1}{i\omega }\nabla \times M+\ldots ,$$

where M is the magnetization vector. The polarization density $P_{g}$ at any point of space depends on the distribution of the macroscopic electric field in a neighborhood of this considered point. As a result, the vector of $P_{g}$ and the spatially averaged induced current contain the effect of the dipolar moment, as well as the effect of all higher-order multipolar moments.

The starting point of our analysis is the power expansion of the nonlocal effective permittivity $\tilde{\epsilon }\left(\omega ,k\right)$ in terms of the small parameter η (the period-to-wavelength ratio) as [36,37]

(2) $$\begin{array}{c} \tilde{\epsilon }\left(\omega ,k\right)=\epsilon_{0}\left(\omega \right)+\eta \beta_{1}\left(\omega \right)+\eta^{2}\beta_{2}\left(\omega \right)+\eta^{3}\beta_{3}\left(\omega \right)+\eta^{4}\beta_{4}\left(\omega \right)+\ldots \\ \;\;\;\;\;\;\;\;\;\;=\epsilon_{0}\left(\omega \right)\left[1+\eta B_{1}\left(\omega \right)+\eta^{2}B_{2}\left(\omega \right)+\eta^{3}B_{3}\left(\omega \right)+\eta^{4}B_{4}\left(\omega \right)+\ldots \right], \end{array}$$

where $\epsilon_{0}$ is the quasistatic (local) permittivity in the limit of k→0, $\eta =d/\lambda_{0}=k_{o}d/2\pi <1,\lambda_{0}=2\pi c/\omega =2\pi /k_{0},\;k_{0}=\omega /c$, and $B_{i}=\beta_{i}/\epsilon_{0}$.

Equation (2) can be derived within the framework of the Landau–Lifshitz approach [38]. It assumes that the induced current density is described in terms of the electric polarization density, while the magnetization is zero. Furthermore, because of the symmetry, $\tilde{\epsilon }\left(\omega ,k\right)=\tilde{\epsilon }\left(\omega ,-k\right)$, that results in the vanishing of the odd-order terms in the expansion (2) [39].

We deal with the fundamental mode, which propagates along the x-direction with the propagation constant $k_{x}=k_{0}\tilde{n}=k_{0}\sqrt{\tilde{\epsilon }}$, where $\tilde{n}$ is the nonlocal effective index of refraction. This mode is in essence a coupled SPP excited at individual interfaces of plasmonic and dielectric layers [40]. The determination of $\tilde{\epsilon }$ is one of the key points of our work.

Generally, the nonlocal effective permittivity can be found using purely numerical techniques, like rigorous coupled-wave analysis, modal method, finite element method, and finite-difference time domain method. Here, we invoke the analytical transfer matrix method [41]. The TMM is a remarkably powerful and versatile tool that can be adapted for studying various microstructures, but it is especially convenient, fruitful, and easy to operate when applied to layered systems, such as superlattices and multilayered waveguides. It can be also applied to finite and infinite periodic multilayers, as well as to 1d graded-index waveguides [42,43].

According to the TMM, if the z-component of the wave vector is zero, the dispersion relation for the the fundamental TM${}_{0}$ mode reads

(3) $$\frac{1}{2}\mathrm{Tr}\left(\prod_{i=1}^{n}M_{i}\right)=1,$$

where

$$M_{i}=\left(\begin{array}{cc} \cos(k_{i}d_{i}) & \frac{\epsilon_{i}}{k_{i}}\sin\left(k_{i}d_{i}\right)\\ -\frac{k_{i}}{\epsilon_{i}}\sin\left(k_{i}d_{i}\right) & \cos(k_{i}d_{i}) \end{array}\right)$$

is the transfer-matrix of the *i*th layer, $\epsilon_{i}$ and $d_{i}$ are its permittivity and thickness, respectively, $k_{i}=\sqrt{k_{0}^{2}\epsilon_{i}-k_{x}^{2}}$ are the *z*-components of the wave vector in the corresponding layers, and *n* is the number of layers in the unit cell. From Equation (3), the nonlocal effective permittivity can be found as

(4) $$\tilde{\epsilon }=\frac{k_{x}^{2}}{k_{0}^{2}}.$$

Equation (3) is, generally speaking, the complex transcendental equation, which can be numerically solved using standard mathematical techniques for both the real $ℜ(\tilde{\epsilon })$ and imaginary $ℑ(\tilde{\epsilon })$ parts of the nonlocal effective permittivity.

In the particular case of n=2, Equation (3) for the TM case reduces to the well-known Rytov equation [44]

(5) $$\cos\left(k_{1}d_{1}\right)\cos\left(k_{2}d_{2}\right)-\frac{1}{2}\left(\frac{\epsilon_{1}k_{2}}{\epsilon_{2}k_{1}}+\frac{\epsilon_{2}k_{1}}{\epsilon_{1}k_{2}}\right)\sin\left(k_{1}d_{1}\right)\sin\left(k_{2}d_{2}\right)=1.$$

In its turn, Equation (5) can be split into two equations,

(6) $$\frac{k_{1}}{\epsilon_{1}}\tan\frac{k_{1}d_{1}}{2}+\frac{k_{2}}{\epsilon_{2}}\tan\frac{k_{2}d_{2}}{2}=0$$

and

(7) $$\frac{k_{1}}{\epsilon_{1}}\tan\frac{k_{2}d_{2}}{2}+\frac{k_{2}}{\epsilon_{2}}\tan\frac{k_{1}d_{1}}{2}=0.$$

Equations (6) and (7) describe the so-called even and odd plasmonic modes, respectively, which correspond to the symmetric and antisymmetric distributions of the local electric field with respect to the middle of layers. The quasistatic term $\epsilon_{0}$ for this case can be derived in the limit of $d_{1},d_{2}\to 0$. This term can be associated with the ED, and it is well known to be

(8) $$\epsilon_{0}=\frac{\epsilon_{1}\epsilon_{2}}{f_{1}\epsilon_{2}+f_{2}\epsilon_{1}},$$

where $f_{1}=d_{1}/d$ and $f_{2}=d_{2}/d$.

As $\eta^{n}\to 0$ in the limit of n→∞, the expansion (2) may be truncated. Having determined $\tilde{\epsilon }$ at several values of the lattice period *d*, this opens a possibility to determine unknown functions $\beta_{i}$ and $B_{i}$ (see Appendix A).

It is commonly accepted that the nonlocal corrections $B_{i}$ are associated with multipole excitations. Their relationship, however, is not straightforward.

## 4. Simulation Results

### 4.1. Material Parameters

In what follows, we take the phase 1 as a transparent isotropic dielectric with the dispersionless permittivity $\epsilon_{1}=2.5$ and the phase 2 as a plasmonic constituent material with the frequency-dependent permittivity

(9) $$\epsilon_{2}\left(\omega \right)=\epsilon_{\infty }-\omega_{p}^{2}/\left(\omega +i\omega \gamma \right).$$

For definiteness, we have set here $\epsilon_{\infty }=4$, the plasma frequency of $\omega_{p}=1.75$ eV, and the collision rate (damping factor) of γ=0.045 eV, that approximately corresponds to zinc oxide heavily doped with aluminum (2 wt%) [45]. On the one hand, such a choice of the Drude model parameters allows one to describe the plasmonic response of ZnO:Al in the telecommunication range of 1.5–2 μm, and on the other hand, if we are dealing with the normalized frequency $\omega /\omega_{p}$ and damping factor $\gamma /\omega_{p}$, it can be considered as typical for doped semiconductors. As to the permittivity of the dielectric phase, its specific choice is not a critical issue because the results of simulations depend on the ratio $\epsilon_{2}/\epsilon_{1}$, but not on the particular values of the permittivities $\epsilon_{1}$ and $\epsilon_{2}$.

Our focus on heavily doped semiconductors is motivated by the fact that, due to lower carrier density, they usually demonstrate a plasmonic resonance peak at lower frequencies (in the IR) as compared to typical metals. This opens up new possibilities for various photonic applications in the IR.

### 4.2. Two-Layer Microgeometry

Let us first consider the simplest case, when the unit cell consists of two layers. An analytical expression for the second-order term $B_{2}$ can be derived using an expansion $\tan\left(z\right)\approx z+z^{3}/3$ for $\tan(\frac{k_{1}d_{1}}{2})$ and $\tan(\frac{k_{2}d_{2}}{2})$ in Equation (6). It is easy to make sure that it is of the form

(10) $$B_{2}=\frac{\pi^{2}}{3}f_{1}^{2}f_{2}^{2}\frac{{\left(\epsilon_{1}-\epsilon_{2}\right)}^{2}}{f_{1}^{3}\epsilon_{2}+f_{2}^{3}\epsilon_{1}}\left[\frac{{\left(f_{1}-f_{2}\right)}^{2}\epsilon_{0}}{f_{1}\epsilon_{2}+f_{2}\epsilon_{1}}+f_{1}f_{2}\right].$$

In the particular case of equal layers ($f_{1}=f_{2}=1/2$), Equation (10) reduces to

(11) $$B_{2}=\frac{\pi^{2}}{24}\frac{{\left(\epsilon_{1}-\epsilon_{2}\right)}^{2}}{\epsilon_{2}+\epsilon_{1}}.$$

The determination of higher-order terms involves cumbersome calculations. Nevertheless, in the case of $f_{1}=f_{2}=1/2$, we have managed to derive an analytical expression for $B_{4}$. It is of the form

(12) $$B_{4}=2B_{2}\frac{\epsilon_{1}^{2}+\epsilon_{2}^{2}}{\epsilon_{2}+\epsilon_{1}}.$$

**Figure 2:**
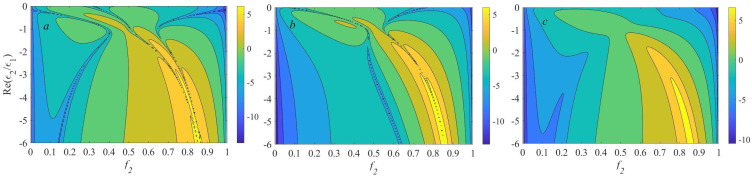
The isolines of (**a**) $\left|ℜ(B_{2})\right|$, (**b**) $\left|ℑ(B_{2})\right|$, and (**c**) $|B_{2}|$, shown as a filled contour plot on the $f_{2}$ − $ℜ(\epsilon_{2}/\epsilon_{1})$ plane, calculated in accordance with Equation (10) (logarithmic scale).

For further analysis, it is useful to study the behavior of the function $B_{2}$. Qualitatively, its dependence on $ℑ(\epsilon_{2})$ is not critically important, and we take here for simplicity $ℑ(\epsilon_{2})=0.3$. The isolines of the absolute value of $ℜ\left(B_{2}\right),\;ℑ\left(B_{2}\right)$ and $B_{2}$ (logarithmic scale) in coordinates of ($f_{2},\;ℜ\left(\epsilon_{2}/\epsilon_{1}\right)$), computed in accordance with Equation (10) are shown in Figure 2. The logarithmic scale allow us to clearly see all zeros and extrema of $ℜ(B_{2})$ and $ℑ(B_{2})$.

As Figure 2 shows, the behavior of the frequency dependence of the second-order correction $B_{2}$ to the nonlocal effective permittivity, despite its simple analytical form, can be nontrivial. The behavior of the higher-order corrections can be even more complicated. Besides, it is necessary to keep in mind that as the plasmonic phase relative content $f_{2}$ becomes large, nonlocality can become strong. If so, the expansion (2) can become inaccurate, or at least more terms in Equation (2) should be retained.

**Figure 3:**
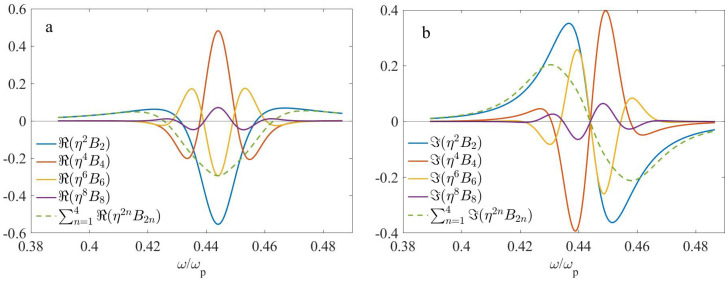
The frequency dependencies of the nonlocal corrections to the effective permittivity at d=150 nm, $f_{1}=0.7,\;f_{2}=0.3$: (**a**) $ℜ(\eta^{2n}B_{2n})$ and (**b**) $ℑ(\eta^{2n}B_{2n})$.

**Figure 4:**
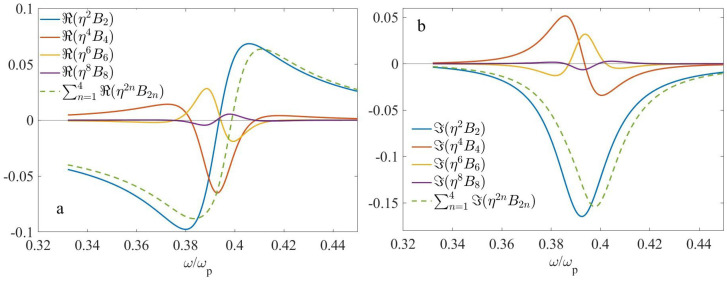
The same as Figure 3, but at $f_{1}=f_{2}=0.5$. (**a**) $ℜ(\eta^{2n}B_{2n})$ and (**b**) $ℑ(\eta^{2n}B_{2n})$.

In the following, we fix the period *d* at d=150 nm, that yields η=d/λ approximately within the range of 0.05–0.1, and $k_{0}d$ approximately within the range of 0.3–0.6. In Figure 3 and Figure 4, we display the nonlocal corrections $\eta^{2}B_{2},\eta^{4}B_{4},\eta^{6}B_{6}$, and $\eta^{8}B_{8}$ for $f_{2}=0.3$ and $f_{2}=0.5$, respectively. The corresponding geometrical parameters are shown in Table 1.

It should be emphasized that the results, shown in Figure 3 and Figure 4, do not depend significantly on the choice of the extraction methods, outlined in Appendix A. In addition, the results for the second-order correction $B_{2}$ and fourth-order correction $B_{4}$, obtained with the use of the above numerical methods, are in good agreement with those obtained with the use of analytical calculations, Equations (11) and (12), respectively. This verifies both the consistency and high accuracy of the numerical procedures used.

### 4.3. Four-Layer Microgeometry

If the unit cell contains 4 layers, there are four parameters (say, $d_{1},d_{2},d_{3}$ and $d_{4}$), which specify microgeometry. If we fix $d=d_{1}+d_{2}+d_{3}+d_{4}$, three of them can be considered as independent. It is obvious that dealing with the full parameter space involves difficulties. Instead of this, we introduce a new geometrical parameter, μ, as $\mu ={\left(d_{1}/d-1/4\right)}^{2}+{\left(d_{2}/d-1/4\right)}^{2}+{\left(d_{3}/d-1/4\right)}^{2}+{\left(d_{4}/d-1/4\right)}^{2}$. This parameter takes its minimal value, μ=0, if $d_{1}/d=d_{2}/d=d_{3}/d=d_{4}/d=1/4$, i.e., for fully symmetric two-layer microgeometry, and its maximum value, μ=0.625, if the relative thickness of any of the layers, $d_{i}/d\to 1$. Thus, in a sense, the parameter μ sets a measure of asymmetry. We are interested in how it can affect the nonlocal effective permittivity in general and various nonlocal corrections $B_{i}$ in particular.

For definiteness, in the paper, we restrict ourselves to the imaginary part of the nonlocal corrections. Doing so, it is necessary to keep in mind that the peaks of the real part are usually correspond to zeros of the imaginary part, and vice versa. For completeness, the corresponding real parts are given in Supplementary Materials, Figures S1–S3.

**Figure 5:**
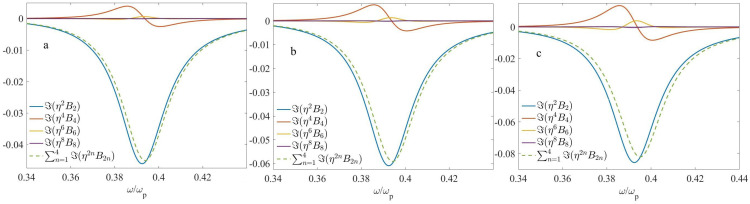
The frequency dependencies of the imaginary part of nonlocal corrections to the effective permittivity at $f_{1}=f_{2}=0.5$: (**a**) $d_{1}/d=0.25,d_{2}/d=0.2,d_{3}/d=0.25,d_{4}/d=0.3$, (**b**) $d_{1}/d=0.25,d_{2}/d=0.15,d_{3}/d=0.25,d_{4}/d=0.35$, and (**c**) $d_{1}/d=0.25,d_{2}/d=0.1,d_{3}/d=0.25,d_{4}/d=0.4$.

By analogy to the two-layer case, we take d=150 nm. As one can expect, three options can be of interest: (1) $f_{2}=1/2$, (2) $f_{2}<1/2$, and (3) $f_{2}>1/2.$ First, in Figure 5, we show the imaginary parts of the nonlocal corrections at $f_{1}=f_{2}=1/2$, computed for three arrangements with different values of $d_{i}/d$. For clarity, the values of the parameters $d_{i}/d$ and corresponding μ are shown in Table 2.

Here, we have fixed the thicknesses of the dielectric layers, $d_{1}$ and $d_{3}$, and varied the thickness of the plasmonic layers, $d_{2}$ and $d_{4}$. Alternatively, we could fix the thicknesses of the plasmonic layers $d_{2}$ and $d_{4}$ and vary $d_{1}$ and $d_{3}$. However, as our computations show, if the parameter μ is constant, the results for $ℑ(B_{i})$ are hardly distinguishable. So, $ℑ(B_{i})$ are almost the same for $d_{1}/d=0.25,d_{2}/d=0.2,d_{3}/d=0.25,d_{4}/d=0.3$ and $d_{1}/d=0.2,d_{2}/d=0.25,d_{3}/d=0.3,d_{4}/d=0.25$ (μ=0.005), $d_{1}/d=0.25,d_{2}/d=0.15,d_{3}/d=0.25,d_{4}/d=0.35$ and $d_{1}/d=0.15,d_{2}/d=0.25,d_{3}/d=0.35,d_{4}/d=0.25$ (μ=0.02), $d_{1}/d=0.25,d_{2}/d=0.1,d_{3}/d=0.25,d_{4}/d=0.4$ and $d_{1}/d=0.1,d_{2}/d=0.25,d_{3}/d=0.4,d_{4}/d=0.25$ (μ=0.045), respectively.

**Figure 6:**
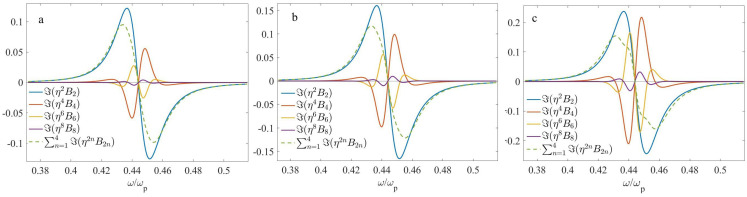
The frequency dependencies of the imaginary part of nonlocal corrections to the effective permittivity at $f_{1}=0.7,\;f_{2}=0.3$: (**a**) $d_{1}/d=0.4,d_{2}/d=0.1,d_{3}/d=0.3,d_{4}/d=0.2$, (**b**) $d_{1}/d=0.5,d_{2}/d=0.1,d_{3}/d=0.2,d_{4}/d=0.2$, and (**c**) $d_{1}/d=0.6,d_{2}/d=0.1,d_{3}/d=0.1,d_{4}/d=0.2$.

Next, in Figure 6, we show the imaginary parts of the nonlocal corrections at $f_{1}=0.7,\;f_{2}=0.3$, computed for three different arrangements. All the geometrical parameters are listed in Table 3.

**Figure 7:**
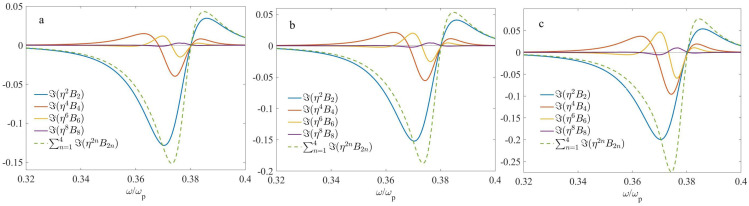
The frequency dependencies of the imaginary part of nonlocal corrections to the effective permittivity at $f_{1}=0.45,\;f_{2}=0.55$: (**a**) $d_{1}/d=0.2,d_{2}/d=0.3,d_{3}/d=0.25,d_{4}/d=0.25$, (**b**) $d_{1}/d=0.2,d_{2}/d=0.35,d_{3}/d=0.25,d_{4}/d=0.2$, and (**c**) $d_{1}/d=0.2,d_{2}/d=0.4,d_{3}/d=0.25,d_{4}/d=0.15$.

Finally, in Figure 7, we show the imaginary parts of nonlocal corrections at $f_{1}=0.45,\;f_{2}=0.55$, computed again for three different arrangements. The parameters of $d_{i}/d$ used in the computations and corresponding values of the parameter μ are shown in Table 4.

## 5. Discussion

First of all, from Equation (8), one can conclude that $ℑ(\epsilon_{0})$ exhibits a Lorentzian profile that is symmetric with respect to the ED resonance, taking place at $f_{1}\epsilon_{2}+f_{2}\epsilon_{1}≃0$, while $ℜ(\epsilon_{0})$ is antisymmetric about this point. At the same time, despite the multipolar origin of all nonlocal corrections $B_{i}$, the peaks and zeros in the spectra of $B_{i}\left(\omega \right)$ cannot be unambiguously associated with specific multipolar excitations.

From Equation (10), one can see that the second-order term $B_{2}$ contains two summands, which can be associated with EQ and MD contributions. The first summand, which originates from the first term in the square brackets of Equation (10), disappears when $f_{1}=f_{2}=1/2$, i.e., for a completely symmetrical arrangement. In contrast, the second summand, which originates from the second term in the square brackets of Equation (10), is always nonzero. Moreover, at $f_{1}=f_{2}=1/2$, it takes its maximum value, given by Equation (11). We identify the first summand as associated with the EQ, while the second summand as associated with the MD. While usually the MD is characterized by an electric displacement loop, it is always present when there is enough retardation (nonuniformity) of the local electric field within the unit cell. As to the EQ moment, it is expected to be zero as a result of high symmetry at $f_{1}=f_{2}=1/2$.

It is obvious that the first term in the square brackets of Equation (10) can be neglected close to the point of $f_{1}=f_{2}=1/2$. In contrast, the first term prevails, while the second term can be neglected well below or well above this point. In the general case, of course, both terms should be taken account. This gives rise to an asymmetry of the $B_{2}\left(\omega \right)$ profile.

Looking at Figure 3 ($f_{1}=0.7,f_{2}=0.3$), we can conclude that $ℜ(B_{i})$ are symmetric, while $ℑ(B_{i})$ are antisymmetric with respect to the point of $\omega /\omega_{p}≃0.4439$, which corresponds to $ℜ(f_{1}\epsilon_{2}+f_{2}\epsilon_{1})≃0$, i.e., to the ED resonance. Near this resonant frequency, the second term in the square brackets of Equation (10) can be neglected. Neglecting the frequency dispersion of $\epsilon_{2}\left(\omega \right)$ in this range, it becomes clear why the second-order term $B_{2}$ exhibits the above symmetry or antisymmetry. Furthermore, the terms $B_{2}$ and $B_{4}$, as well as $B_{6}$ and $B_{8}$ have opposite signs and hence they compensate each other. Because of the compensation effect, $\tilde{\epsilon }≃\epsilon_{0}+\eta^{2}B_{2}+\eta^{4}B_{4}$ might be a more accurate approximation for this case than $\tilde{\epsilon }≃\epsilon_{0}+\eta^{2}B_{2}+\eta^{4}B_{4}+\eta^{6}B_{6}$. Ultimately, although the particular terms $B_{i}$ can be rather large, resulted nonlocality is moderate.

According to Figure 4 ($f_{1}=f_{2}=0.5$), the zeros of $ℜ(B_{2})$, $ℜ(B_{6})$, $ℜ(B_{8})$, as well as the minimum of $ℜ(B_{4})$ take place at approximately the same point, $\omega /\omega_{p}≃0.394$, that is close to the ED resonance, which occurs at $\epsilon_{2}≃-\epsilon_{1}$. The frequency dependence of $ℑ(B_{2})$ near this point is negative and reaches its minimum, in accordance with Equation (11). $ℜ\left(B_{2}\right),ℜ\left(B_{6}\right),ℜ\left(B_{8}\right)$ and $ℑ(B_{4})$ exhibit antisymmetry, while $ℑ\left(B_{2}\right),ℑ\left(B_{6}\right),ℑ\left(B_{8}\right)$ and $ℜ(B_{4})$ exhibit symmetry with respect to the above point. Overall, nonlocality is relatively weak for this case; that can be related to the disappearance of the EQ contribution.

When $f_{2}$ is well above 1/2 (this case is not shown here), $ℑ(B_{2})$ is antisymmetric with respect to the ED resonance frequency; the position of its zero corresponds to a zero of $ℜ(\epsilon_{0})$ or the maximum of $ℑ(\epsilon_{0})$. At the same time, $B_{2}$ then can take large values and nonlocality is strong, that restricts the use of the expansion (2).

Let us now consider the four-layer case. At $f_{1}=f_{2}=0.5$ (Figure 5), the behavior of all $B_{i}$ is qualitatively similar to that of the two-layer case (Figure 4). At the same time, nonlocality is weaker as a whole. All nonlocal corrections rise with μ; moreover, higher-order terms $B_{4},B_{6}$ and $B_{8}$ rise faster than $B_{2}$.

By analogy with the previous case, at $f_{1}=0.7,f_{2}=0.3$ (Figure 6), the behavior of the nonlocal corrections is qualitatively similar to that in Figure 4. The fast rise of the higher-order corrections with the parameter μ should be noticed.

Finally, we invoke Figure 7 to analyze the behavior of the nonlocal corrections $B_{i}$ at $f_{1}=0.45,\;f_{2}=0.55$. As can be seen, zeros of $ℑ(B_{i})$ take place at approximately the same frequency, $\omega /\omega_{p}≃0.38$. It is easy to check that this is close to the ED resonance. At this point, the contribution of the nonlocal corrections to $ℑ(\tilde{\epsilon })$ is about zero. In contrast, their contribution to $ℜ(\tilde{\epsilon })$ (not shown here) is maximal. All $B_{i}\left(\omega \right)$ spectra become strongly asymmetric with respect to the ED resonance frequency. As one can expect, this is because the taken value of $f_{2}$ is close to 1/2; hence, both terms should be accounted for in Equation (10). Again, all the nonlocal corrections tend to rise with the parameter μ.

In general, the higher the term $B_{i}$ is, the more oscillations appear. For example, when $ℑ(B_{2})$ has only one zero, $ℑ(B_{4})$ has two ones, and $ℑ(B_{6})$ has five zeros. This means that more multipoles are coming into the game for every next nonlocal correction $B_{i}$.

Overall, if the unit cell period *d* is fixed, nonlocality is weaker for four-layer microgeometries as compared to two-layer ones. This is consistent with the fact that all layers within the unit cell become thinner; hence, MM becomes “more homogeneous”.

All the nonlocal terms $B_{i}$ could be controlled by tuning both material and geometrical parameters. For example, according to Equation (12), at $f_{1}=f_{2}=1/2$, the $B_{4}$ term becomes especially large near the ED resonance in the case of high dielectric contrast. Currently, various techniques are known to tune the permittivity of constituents, say, using applied electric field [46], magnetic field [47], or temperature [48].

## 6. Conclusions

In this paper, we have presented a straightforward method to retrieve the nonlocal corrections to the effective permittivity of 1d plasmonic two-phase MMs with two-layer and four-layer unit cells under TM polarization. This opens up an avenue to analyze the spectrum of multipolar excitations in 1d MMs. Our study shows that MMs with such simple microgeometries are fraught with many surprises and offer many opportunities for tailoring their diverse functionalities. The key findings of our study can be formulated as follows:

(1) When the dielectric phase content prevails ($f_{1}>f_{2}$), particular nonlocal terms $B_{i}$ can be large, but overall nonlocality can be moderate due to their mutual compensation.
(2) When $f_{1}=f_{2}=1/2$, symmetry of the $B_{4}$ term is opposite to that of other nonlocal contribution terms under consideration.
(3) When the plasmonic phase content prevails ($f_{1}<f_{2}$), strong nonlocality can occur.
(4) The spectra of multipolar excitations of the MMs with four-layer unit cell reproduce main features of MMs with two-layer unit cell, but nonlocality in the former case is weaker.
(5) Higher asymmetry in four-layer arrangements enhances nonlocality.

This study provides information that could be useful for the design of nanomaterials and nanostructures with extraordinary optical properties. As we expect, it could have an impact on the quantitative modeling in typical applications and, in turn, could allow for developing nano-optical devices with unique functionalities.

## Figures and Tables

**Table 1:** The geometrical parameters which correspond to Figure 3 and Figure 4.

	$f_{1}$	$f_{2}$	*d*, nm
Figure 3	0.7	0.3	150
Figure 4	0.5	0.5	150

**Table 2:** The geometrical parameters which correspond to Figure 5.

$f_{2}$	$d_{1}/d$	$d_{2}/d$	$d_{3}/d$	$d_{4}/d$	μ	Label
	0.25	0.2	0.25	0.3	0.005	a
0.5	0.25	0.15	0.25	0.35	0.02	b
	0.25	0.1	0.25	0.4	0.045	c

**Table 3:** The geometrical parameters which correspond to Figure 6.

$f_{2}$	$d_{1}/d$	$d_{2}/d$	$d_{3}/d$	$d_{4}/d$	μ	Label
	0.4	0.1	0.3	0.2	0.05	a
0.3	0.5	0.1	0.2	0.2	0.09	b
	0.6	0.1	0.1	0.2	0.17	c

**Table 4:** The geometrical parameters that correspond to Figure 7.

$f_{2}$	$d_{1}/d$	$d_{2}/d$	$d_{3}/d$	$d_{4}/d$	μ	Label
	0.2	0.3	0.25	0.25	0.005	a
0.55	0.2	0.35	0.25	0.2	0.015	b
	0.2	0.4	0.25	0.15	0.035	c

## Data Availability

The data presented in this study are available on request from the corresponding author.

## Supplementary Materials

The following supporting information can be downloaded at: https://www.mdpi.com/article/10.3390/nano13081395/s1. Figure S1: The same as Figure 5, but for the corresponding real parts. Figure S2: The same as Figure 6, but for the corresponding real parts. Figure S3: The same as Figure 7, but for the corresponding real parts.

## Abbreviations

The following abbreviations are used in this manuscript:

ED	electric dipole
MD	magnetic dipole
EQ	electric quadrupole
MQ	magnetic quadrupole
EO	electric octopole
MM	Metamaterial
SPP	surface plasmon polariton
TMM	transfer matrix method
IR	infrared
1d	one-dimensional

## Appendix A

Discarding in Equation (2) terms of the order of $\eta^{10}$ and higher, one has the following system of linear equations:

(A1) $$\left\{\begin{array}{c} \eta_{1}^{2}\beta_{2}\left(\omega \right)+\eta_{1}^{4}\beta_{4}\left(\omega \right)+\eta_{1}^{6}\beta_{6}\left(\omega \right)+\eta_{1}^{8}\beta_{8}\left(\omega \right)=\Delta_{1}\left(\omega \right),\\ \eta_{2}^{2}\beta_{2}\left(\omega \right)+\eta_{2}^{4}\beta_{4}\left(\omega \right)+\eta_{2}^{6}\beta_{6}\left(\omega \right)+\eta_{2}^{8}\beta_{8}\left(\omega \right)=\Delta_{2}\left(\omega \right),\\ \eta_{3}^{2}\beta_{2}\left(\omega \right)+\eta_{3}^{4}\beta_{4}\left(\omega \right)+\eta_{3}^{6}\beta_{6}\left(\omega \right)+\eta_{3}^{8}\beta_{8}\left(\omega \right)=\Delta_{3}\left(\omega \right),\\ \eta_{4}^{2}\beta_{2}\left(\omega \right)+\eta_{4}^{4}\beta_{4}\left(\omega \right)+\eta_{4}^{6}\beta_{6}\left(\omega \right)+\eta_{4}^{8}\beta_{8}\left(\omega \right)=\Delta_{4}\left(\omega \right), \end{array}\right.\;$$

where $\eta_{i}=k_{o}D_{i}/2\pi ,\;\Delta_{i}\left(\omega ,D_{i}\right)=\tilde{\epsilon }\left(\omega \right)-\epsilon_{0}\left(\omega \right).$ Doing so, we suppose that the $\eta_{i}^{8}\beta_{8}$ term absorbs the remainder.

The above system can be solved for the unknown functions $\beta_{i}$ using a standard procedure of matrix inversion:

(A2) $$\left(\begin{array}{c} \beta_{2}\\ \beta_{4}\\ \beta_{6}\\ \beta_{8} \end{array}\right)=A^{-1}\left(\begin{array}{c} \Delta_{1}\\ \Delta_{2}\\ \Delta_{3}\\ \Delta_{4} \end{array}\right),$$

where $A^{-1}$ is the inverse of the matrix

$$A=\left(\begin{array}{cccc} \eta_{1}^{2} & \eta_{1}^{4} & \eta_{1}^{6} & \eta_{1}^{8}\\ \eta_{2}^{2} & \eta_{2}^{4} & \eta_{2}^{6} & \eta_{2}^{8}\\ \eta_{3}^{2} & \eta_{3}^{4} & \eta_{3}^{6} & \eta_{3}^{8}\\ \eta_{4}^{2} & \eta_{4}^{4} & \eta_{4}^{6} & \eta_{4}^{8} \end{array}\right).$$

It is necessary to keep in mind that the above procedure may become inaccurate if det $(A^{-1})\ll 1$. In this case, the quantities $\beta_{2},\beta_{4},\beta_{6}$, and $\beta_{8}$ can be found by sequentially taking the limits

(A3) $$\beta_{2}=\lim_{\eta \to 0}\frac{\Delta }{\eta^{2}},$$

(A4) $$\beta_{4}=\lim_{\eta \to 0}\frac{\Delta /\eta^{2}-\beta_{2}}{\eta^{2}},$$

(A5) $$\beta_{6}=\lim_{\eta \to 0}\frac{\Delta /\eta^{2}-\beta_{2}-\eta^{2}\beta_{4}}{\eta^{4}},$$

(A6) $$\beta_{8}=\lim_{\eta \to 0}\frac{\Delta /\eta^{2}-\beta_{2}-\eta^{2}\beta_{4}-\eta^{4}\beta_{6}}{\eta^{6}}.$$
