# Supplementary material for: Multipole Excitations and Nonlocality in 1d Plasmonic Nanostructures
Source: Nanomaterials (Basel). 2023 Apr 18;13(8):1395. doi: 10.3390/nano13081395 (PMC10144308; doi:10.3390/nano13081395)
Supplement: Supplementary file 1 [file nanomaterials-13-01395-s001.zip › nanomaterials-2334160-supplementary.pdf]

Supplementary Materials

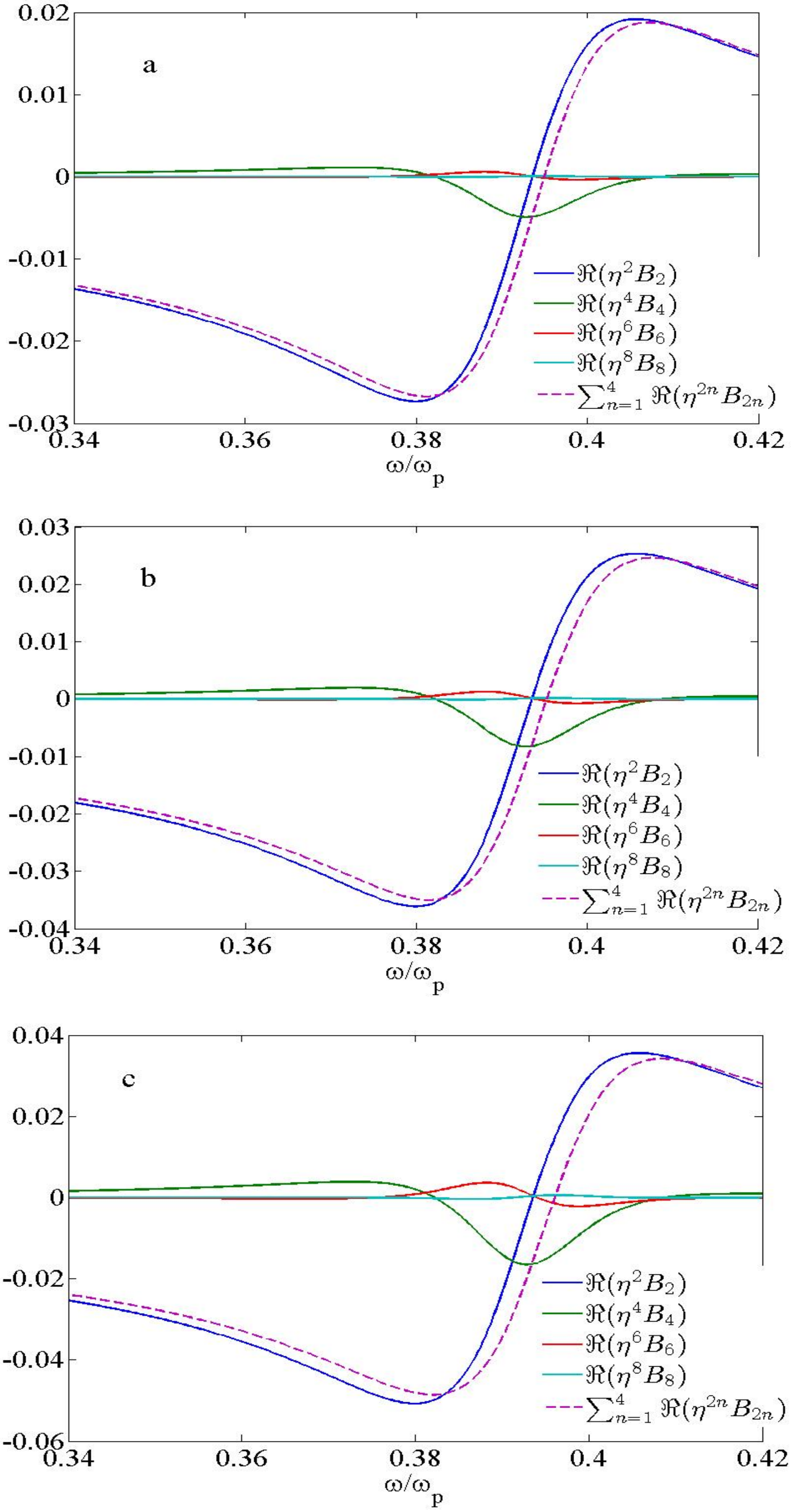

Figure S1. The real parts of the nonlocal corrections to the effective permittivity at f_1=f_2=0.5. The parameters of the unit cells are given in Table 2.

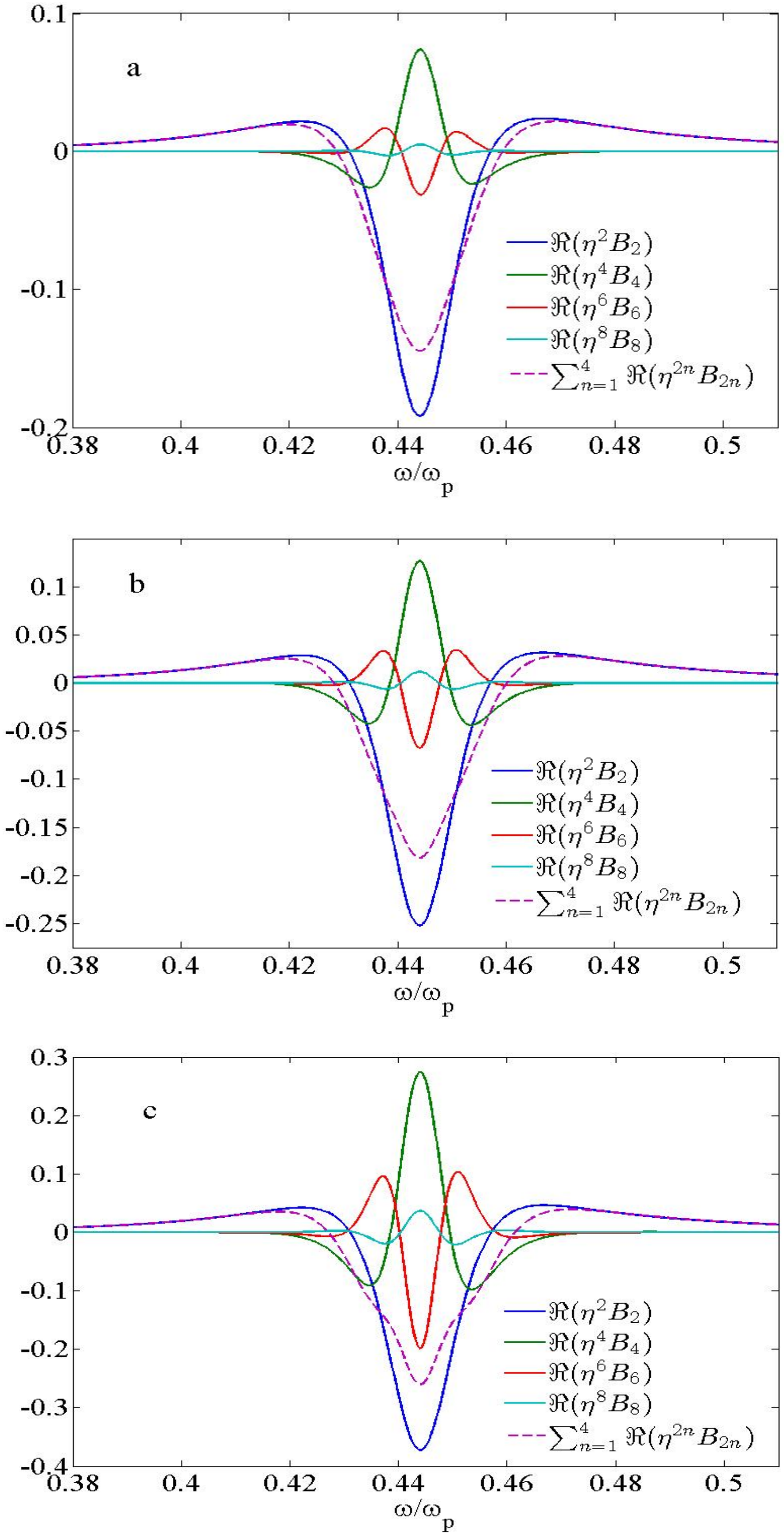

Figure S2. The real parts of the nonlocal corrections to the effective permittivity at f_1=0.7, f_2=0.3. The parameters of the unit cells are given in Table 3.

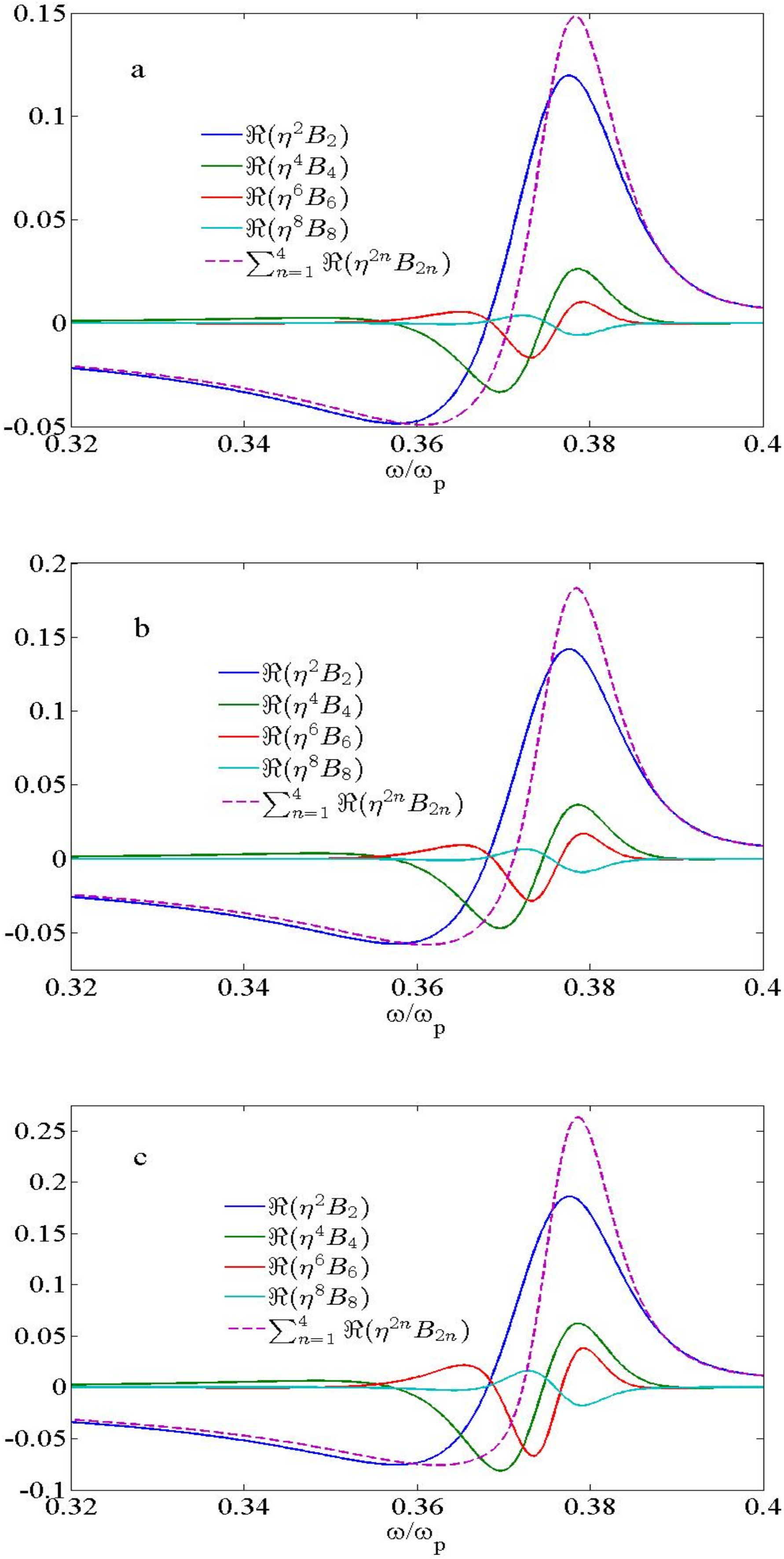

Figure S3. The real parts of the nonlocal corrections to the effective permittivity at f_1=0.45, f_2=0.55. The parameters of the unit cells are given in Table 4.
